# Nutritional profile of plant-based dairy alternatives in the Swedish market

**DOI:** 10.1016/j.crfs.2024.100712

**Published:** 2024-03-13

**Authors:** Hanieh Moshtaghian, Elinor Hallström, Marta Bianchi, Susanne Bryngelsson

**Affiliations:** a Department of Agriculture and Food, Research Institutes of Sweden (RISE), Gothenburg, Sweden; b Department of Agriculture and Food, Research Institutes of Sweden (RISE), Lund, Sweden

**Keywords:** Dairy analogues, Milk alternatives, Organic products, Nutritional quality, Alternative proteins, Sustainable diet

## Abstract

The market for plant-based dairy alternatives is growing; therefore, focusing on the nutritional quality of these products is important. This study evaluates the nutritional profile of plant-based alternatives to milk, yoghurt, cheese, cream, ice cream and fat spread in the Swedish market and compares them to corresponding dairy products. The nutritional quality of organic vs non-organic and plain vs flavoured plant-based milk and yoghurt alternatives was also assessed. Nutritional data for 222 plant-based dairy alternatives were collected from the manufacturers’ websites, and data for corresponding dairy products were obtained from the Swedish Food Composition Database. Plant-based dairy alternatives had higher fibre content than dairy products, while their protein content was lower, except for soy-based products. The saturated fat content of plant-based dairy alternatives was similar to or lower than dairy products, except for coconut-based yoghurt and plant-fat-based cheese. Their energy content was also similar to or lower than dairy products, except for coconut-based yoghurt, plant-based fat spread and plant-based ice cream, which contained higher energy than yoghurt, blended margarine, and ice cream, respectively. The micronutrient fortification was mainly in plant-based milk, yoghurt, and cheese alternatives; thus, compared to dairy, they had similar or higher vitamins D, B2, and B12 (except in plant-based milk alternatives), calcium and iodine content. Furthermore, organic plant-based milk and yoghurt alternatives had a lower micronutrient content (e.g., vitamins B2 and B12, iodine and calcium) except for vitamin D than non-organic varieties. Flavoured plant-based milk and yoghurt alternatives were higher in energy and total sugar than plain varieties. In summary, plant-based dairy alternatives have nutritional strengths and weaknesses compared to dairy products that should be considered when replacing dairy.

## Highlights

• Dairy alternatives had higher fibre and lower protein contents than dairy.
• Flavoured milk/yoghurt alternatives had higher energy and sugar than plain products.
• Fortified dairy alternatives had similar or higher micronutrient content than dairy.
• Organic vs non-organic dairy alternatives had fewer micronutrients except vitamin D.

## Graphical abstract

**Figure:**
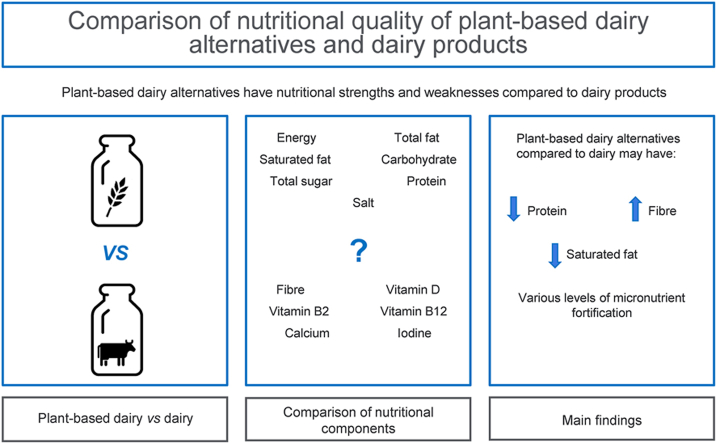

## 1 Introduction

Plant-based dairy alternatives are known as dairy analogues, imitation dairy products, dairy substitutes, and dairy mimics (Muthukrishnan, 2021). These alternatives may be consumed as a substitute for conventional bovine milk-based products, particularly for those who avoid dairy. Approximately 65% of the world population and 10 % of Swedes are lactose intolerant (Bayless et al., 2017). Many people also avoid dairy-based products for other reasons such as milk protein allergies, hypercholesterolemia, lifestyle choices (vegan and vegetarianism), affordability, ethical reasons (animal welfare), and environmental sustainability (Mäkinen et al., 2016; Muthukrishnan, 2021; Sethi et al., 2016). In recent years, consumer demands for plant-based dairy alternatives have increased, and in 2022, the global market for these products was valued at USD 27.5 billion (Pulidindi and Ahuja, 2023). The global compound annual growth rate for plant-based dairy alternatives is estimated to rise 9% by 2032 (Pulidindi and Ahuja, 2023).

As the market and intake of plant-based dairy alternatives increase, so does the importance of their nutritional contribution to dietary intake. These alternatives usually contain various plant-based ingredients to replace dairy's animal-sourced protein and fat (Sołowiej and Nastaj, 2016). Thus, the nutritional quality of plant-based dairy alternatives depends on the types of ingredients, processing status, and micronutrient fortification (Silva et al., 2020). The main ingredients for substituting milk components in these products are nuts, seeds, grains, legumes, and coconut (Craig and Fresán, 2021; Chalupa-Krebzdak et al., 2018; Singhal et al., 2017). Since the nutritional content of these products varies (Craig and Fresán, 2021), they can be considered a more or less healthy alternative to dairy (Fresán and Rippin, 2021).

Dairy products are one of the common food items in a daily diet. In Sweden, the average milk and yoghurt intake is 245 g/day, and the average cheese intake is 25 g/day (Swedish National Food Agency, 2012). These products are important dietary sources of several macro- and micronutrients (Swedish National Food Agency, 2012), including protein, fat, calcium, iodine, and vitamins B2 and B12 (Kanekanian, 2014). Therefore, plant-based dairy alternatives that substitute milk and other milk-derived products should have an identical composition to fulfil a similar dietary role as dairy products. However, scientific studies do not fully support this perception; for example, some studies express concern regarding the lower intake of some of the macro- and micronutrients, including protein, calcium, and vitamin B12, when replacing milk with plant-based milk alternatives (Clegg et al., 2021; Zhang et al., 2020; Chalupa-Krebzdak et al., 2018). On the other hand, plant-based milk alternatives have some nutritional benefits over milk, such as lower saturated fat and higher fibre content (Clegg et al., 2021). To understand the impact of replacing dairy with plant-based dairy alternatives in the diet, it is crucial to constantly assess the nutritional quality of these products in the dynamic market and compare them with dairy.

To our knowledge, most studies investigated the nutritional quality of plant-based milk alternatives (Smith et al., 2022; Singhal et al., 2017), and few studies assessed the nutrient content of other plant-based dairy alternatives, such as yoghurt and cheese (Craig and Brothers, 2021; Craig et al., 2022; Clegg et al., 2021). Studies regarding the nutritional quality of plant-based alternatives to cream, ice cream (Tonheim et al., 2022), and fat spreads are also scarce. Furthermore, none of the studies was conducted in the Swedish market. Thus, further investigation in this context is warranted due to the limited availability of studies on the nutritional quality of various plant-based dairy alternatives in the market, particularly the Swedish market. This type of information can assist manufacturers in considering the advantages and disadvantages of plant-based dairy alternatives in product development. It can also help consumers and dietitians account for these strengths and limitations when suggesting the replacement of dairy products with plant-based dairy alternatives.

Therefore, the aim of this study is to assess the nutritional profile of plant-based alternatives to milk, yoghurt, cheese, cream, ice cream, and fat spreads available in the Swedish market and compare them to corresponding dairy products. Additionally, the nutritional quality of organic vs non-organic and plain vs flavoured plant-based milk and yoghurt alternatives was evaluated.

## 2 Material and methods

### 2.1 Data collection for plant-based dairy alternatives

The brands of plant-based dairy alternatives were identified through the main Swedish supermarkets' (ICA and Coop) websites. The brands under plant-based, dairy-free, vegetarian, and allergy sections were considered. To be classified as plant-based dairy alternatives, these products should not contain milk, casein, caseinates, whey proteins, and milk fat (butter and anhydrous milk fat) in their ingredient list. Data for 222 products were collected between April and November 2022 from the manufacturers’ websites. Data were imputed from the retailers’ websites, other online shops, and product nutrition labels if any inconsistencies were identified. Collected data included ingredients, energy, and nutrients that were mandatory to declare (total fat, saturated fat, total carbohydrate, total sugar, protein, and salt) as well as voluntary to declare (e.g., fibre, vitamin D, vitamin B2, vitamin B12, calcium, and iodine) per 100 g. Thus, all plant-based dairy alternatives had energy and mandatory-to-declare nutrients data, but values on voluntary-to-declare nutrients were not available for all products.

Product descriptions on the manufacturers’ websites were considered for food grouping. Plant-based Milk Alternatives (PBMAs) were products identical to plain and flavoured milk, excluding coffee-based drinks. Canned coconut milk intended for cooking was considered in the cream food group. Plant-based Yoghurt Alternatives (PBYAs) were products similar to plain and flavoured yoghurts, and Plant-based Cheese Alternatives (PBChAs) were plain and flavoured alternatives to hard and soft cheeses. Plant-based Cream Alternatives (PBCrAs) were alternatives to cream, cooking cream, whipped cream, crème fraîche, and sour cream. Plant-based Ice cream Alternatives (PBIAs) were all plant-based ice creams except the coated (fruit ice and cocoa couverture) and cone ice creams. Plant-based Fat Spread Alternatives (PBSAs) included plant-based solid and liquid margarine, excluding blended animal- and plant-based varieties.

PBMAs and PBYAs were compared to the corresponding dairy products based on their main dairy substitute ingredient. For most items, the manufacturer declared the main plant-based ingredient; however, if this was unclear, the main ingredient was identified from the ingredient list. Organic, non-organic, plain, and flavoured PBMAs and PBYAs were also grouped according to manufacturer declaration. The main ingredients of PBChAs and PBCrAs were categorised based on plant fats and the combination of plant fat and protein. Therefore, foods from these groups were classified into plant fat-based and plant fat- and protein-based products. The nutritional profile of PBIAs and PBSAs were assessed without considering any subgroups due to ingredient diversity.

### 2.2 Selection of dairy products for comparison

Corresponding dairy products were selected from the Swedish Food Composition Database (Swedish Food Agency, 2022) for comparison with plant-based dairy alternatives. Plain and flavoured milk and yoghurt products, regardless of their fat content, were considered for comparison with PBMAs and PBYAs, respectively. It is worth mentioning that fermented milk products not intended for drinking, such as filmjölk, A-fil and långfil, were considered in the yoghurt group. Soft, hard, and processed cheeses with various fat content were compared to PBChAs. Different types of cream and ice cream (excluding fruit ice and cocoa couverture, and cone ice cream) with diverse fat ranges were compared to PBCrAs and PBIAs, respectively. Butter and blended animal- and plant-based margarine were also compared to PBSAs.

### 2.3 Statistical analyses

Energy and nutrients, mandatory and voluntary to declare, were presented as median (min., max.). The proportion of plant-based dairy alternatives fortified with vitamins D, B2, B12, calcium, and iodine were also calculated and expressed as percentages. The normality of data was tested by the Shapiro-Wilk test and rejected; thus, plant-based dairy alternatives were compared to corresponding dairy products using the Mann-Whitney *U* test. For ingredient-based comparisons — if only one product was available — no statistical analysis was conducted to compare the product with the corresponding dairy product. Further comparisons were also made for organic vs non-organic and plain vs flavoured PBMAs and PBYAs using the Mann-Whitney *U* test. Statistical analyses were conducted in SPSS (Version 25, SPSS Inc., Chicago, IL), and statistical significance was set at P_value_ <0.05.

## 3 Results

### 3.1 Nutritional profile of plant-based dairy alternatives

The energy and nutrient contents of plant-based dairy alternatives are presented in Table 1. The median energy content ranged from 42 kcal/100 g (PBMAs) to 681 kcal/100 g (PBSAs), and the median fat content ranged from 1.5 g/100 g (PBMAs) to 75 g/100 g (PBSAs). Median protein content was between 0 and 3.3 g/100 g, with PBSAs at the lowest end and PBYAs at the highest end of this range. Median salt content was the highest in the PBChAs (1.9 g/100 g) and lowest in PBMAs and PBYAs (0.1 g/100 g). For some products that voluntarily declared fibre, the median fibre content was between 0 g and 6.1 g/100 g in PBSAs and PBChAs, respectively. Regarding those products that declared micronutrients, the median vitamin D and B2 contents were the highest in PBSAs compared to other food groups. Moreover, the median vitamin B12 content in PBChAs and PBSAs (2.5 mcg/100 g) was higher than in other food groups. The median calcium and iodine contents of PBChAs that declared these nutrients were also higher (calcium: 650 mg/100 g and iodine:87 mg/100 g) than other groups.

**Table 1:** Median (min., max.) of energy and nutrients in 100 g plant-based dairy alternatives.

	PBMAs (n = 73)	PBYAs (n = 41)	PBChAs (n = 36)	PBCrAs (n = 22)	PBIAs (n = 23)	PBSAs (n = 27)
*Energy and nutrients (mandatory to declare)*						
Energy (kcal)	42 (13, 74)	75 (41, 179)	286 (213, 628)	169 (80, 420)	232 (169, 276)	681 (315, 900)
Total fat (g)	1.50 (0.50, 3.00)	2.20 (0.90, 18.00)	23.00 (13.00, 68.00)	15.00 (7.00, 28.00)	11.00 (4.50, 15.00)	75.00 (35.00, 100.00)
Saturated fat (g)	0.30 (0.00, 1.40)	0.30 (0.00, 16.00)	18.00 (2.50, 26.00)	9.15 (0.90, 26.00)	6.60 (1.20, 10.50)	20.00 (6.80, 91.00)
Carbohydrate (g)	4.40 (0.00, 12.00)	9.20 (0.00, 16.00)	21.00 (3.30, 32.00)	5.95 (0.00, 47.00)	31.00 (24.00, 37.60)	0.00 (0.00, 4.00)
Total sugar (g)	2.70 (0.00, 11.00)	7.40 (0.00, 10.10)	0.50 (0.00, 3.60)	2.90 (0.00, 44.00)	22.00 (13.90, 28.00)	0.00 (0.00, 2.90)
Protein (g)	1.00 (0.00, 6.00)	3.30 (0.60, 5.80)	0.50 (0.00, 7.00)	1.00 (0.00, 3.50)	1.60 (0.50, 3.60)	0.00 (0.00, 0.50)
Salt (g)	0.10 (0.01, 0.35)	0.10 (0.03, 0.36)	1.90 (0.71, 3.50)	0.11 (0.01, 1.20)	0.14 (0.00, 0.35)	1.00 (0.00, 1.70)
*Selected nutrients (non-mandatory to declare)*						
Fibre (g)^a^	0.60 (0.00, 1.50)	0.90 (0.00, 2.60)	6.10 (0.80, 6.10)	0.60 (0.40, 1.00)	1.10 (0.70, 1.40)	0.00
Vitamin D (mg)^b^	0.75 (0.75, 1.50)	1.00 (0.60, 1.50)	4.00 (4.00, 4.00)	1.00	N/A	20.00 (6.80, 20.00)
Vitamin B2 (mg)^c^	0.21 (0.20, 0.21)	0.21 (0.21, 0.21)	0.90 (0.90, 0.90)	0.21	N/A	1.40 (1.40, 1.40)
Vitamin B12 (mcg)^d^	0.38 (0.38, 0.60)	0.38 (0.30, 0.60)	2.50 (0.50, 2.50)	0.40	N/A	2.50 (2.50, 2.50)
Calcium (mg)^e^	120 (10, 120)	120 (14, 131)	650 (120, 650)	18 (10, 120)	N/A	N/A
Iodine (mg)^f^	22.50 (16.00, 23.00)	22.50 (22.50, 25.00)	87.00 (87.00, 87.00)	22.50	N/A	N/A

PBMAs: Plant-based Milk Alternatives; PBYAs: Plant-based Yoghurt Alternatives; PBChAs: Plant-based Cheese Alternatives; PBCrAs: Plant-based Cream Alternatives; PBIAs: Plant-based Ice-cream Alternatives; PBSAs: Plant-based fat Spread Alternatives; N/A: data not available.

a Number of plant-based products reporting dietary fibre content: PBMAs (n = 52), PBYAs (n = 25), PBChAs (n = 3), PBCrAs (n = 9), PBIAs (n = 5), PBSAs (n = 1).

b Number of plant-based products reporting vitamin D content: PBMAs (n = 59), PBYAs (n = 34), PBChAs (n = 4), PBCrAs (n = 1), PBSAs (n = 22).

c Number of plant-based products reporting vitamin B2 content: PBMAs (n = 48), PBYAs (n = 24), PBChAs (n = 3), PBCrAs (n = 1), PBSAs (n = 2).

d Number of plant-based products reporting vitamin B12 content: PBMAs (n = 53), PBYAs (n = 30), PBChAs (n = 30), PBCrAs (n = 1), PBSAs (n = 2).

e Number of plant-based products reporting calcium content: PBMAs (n = 61), PBYAs (n = 31), PBChAs (n = 10), PBCrAs (n = 5).

f Number of plant-based products reporting iodine content: PBMAs (n = 14), PBYAs (n = 8), PBChAs (n = 4), PBCrAs (n = 1).

### 3.2 Fortification of plant-based dairy alternatives

The percentage of fortified plant-based dairy alternatives is shown in Table 2. More than 80% of PBMAs, PBYAs and PBSAs and 11% of PBChAs were fortified with vitamin D. Vitamin B2 fortification occurred in around 60% of PBMAs and PBYAs. Approximately 70% of PBMAs and PBYAs and over 80% of PBChAs were fortified with vitamin B12. Calcium fortification was observed in 79% of PBMAs, 73% of PBYAs, and 28% of PBChAs. Iodine fortification was not common, and less than 20% of PBMAs and PBYAs and 11% of PBChAs were fortified with iodine. One of the PBCrAs was fortified with vitamins D, B2, B12, and iodine, and two of them were fortified with calcium. None of the PBIAs were fortified.

**Table 2:** Number (percentage) of fortified plant-based dairy alternatives.

	PBMAs (n = 73)	PBYAs (n = 41)	PBChAs (n = 36)	PBCrAs (n = 22)	PBIAs (n = 23)	PBSAs (n = 27)
Vitamin D, n (%)	59 (80.82)	34 (82.93)	4 (11.11)	1 (4.55)	N/A	22 (81.48)
Vitamin B2, n (%)	48 (65.75)	24 (58.54)	3 (8.33)	1 (4.55)	N/A	2 (7.41)
Vitamin B12, n (%)	53 (72.60)	30 (73.17)	30 (83.33)	1 (4.55)	N/A	2 (7.41)
Calcium, n (%)	58 (79.45)	30 (73.17)	10 (27.78)	2 (9.09)	N/A	N/A
Iodine, n (%)	14 (19.18)	8 (19.51)	4 (11.11)	1 (4.55)	N/A	N/A

PBMAs: Plant-based Milk Alternatives; PBYAs: Plant-based Yoghurt Alternatives' PBChAs: Plant-based Cheese Alternatives; PBCrAs: Plant-based Cream Alternatives; PBIAs: Plant-based Ice-cream Alternatives; PBSAs: Plant-based fat Spread Alternatives; N/A: data not available.

### 3.3 Nutritional profile of plant-based milk and yoghurt alternatives

PBMAs’ main ingredients were oats, soybeans, almonds, coconut, rice, peas, potatoes, cashew nuts, and broad beans. Some products contained a combination of these ingredients, i.e., soybeans and peas, soybeans and coconut, coconut and almonds, and coconut and rice. Table 3 provides information on the energy and nutrient contents of PBMAs according to the main ingredients and compares them to milk (data on one cashew nut- and one broad bean-based milk alternative are shown in Appendix A).

**Table 3:** Median (min., max.) of energy and nutrients in 100 g PBMAs (Plant-based Milk Alternatives) according to main milk substitute ingredient and comparison with milk^a^.

	Oat (n = 24)	Soybean (n = 15)	Almond (n = 8)	Coconut (n = 2)	Rice (n = 5)	Pea (n = 5)	Potato (n = 3)	Mixed^b^ (n = 9)	Milk (n = 7)
*Energy and nutrients (mandatory to declare)*									
Energy (kcal)	52 (37, 74)	40 (28, 69)	18 (13, 24)**	17 (14, 20)*	49 (22, 50)	35 (25, 52)	39 (39, 53)	24 (20, 74)	50 (35, 70)
Total fat (g)	1.50 (0.50, 3.00)	1.90 (1.20, 2.90)	1.20 (1.10, 1.40)	1.40 (1.20, 1.60)	1.00 (0.50, 1.10)	2.10 (2.00, 3.00)	3.00 (1.50, 3.00)	0.90 (0.80, 3.00)	1.50 (0.05, 4.20)
Saturated fat (g)	0.20 (0.10, 0.50)*	0.30 (0.20, 0.60)*	0.10 (0.10, 0.50)*	1.25 (1.10, 1.40)	0.10 (0.00, 0.10)*	0.30 (0.20, 0.30)	0.20 (0.10, 0.20)	0.80 (0.50, 0.90)	1.00 (0.00, 2.70)
Carbohydrate (g)	6.70 (5.60, 12.00)**	2.50 (0.00, 6.20)*	0.85 (0.00, 2.60)**	0.45 (0.00, 0.90)*	9.90 (3.90, 10.00)	2.00 (0.35, 5.10)*	4.40 (1.30, 4.80)	2.70 (2.50, 11.00)	4.80 (4.70, 9.80)
Total sugar (g)	4.10 (0.00, 11.00)	2.50 (0.00, 6.20)**	0.35 (0.00, 2.50)**	0.10 (0.00, 0.20)*	3.30 (0.00, 7.10)	2.00 (0.00, 4.90)*	1.80 (0.10, 1.80)*	2.50 (1.90, 7.50)*	4.90 (4.70, 8.80)
Protein (g)	1.00 (0.20, 1.40)***	3.30 (2.10, 5.00)	0.50 (0.40, 0.70)**	0.10 (0.10, 0.10)*	0.10 (0.00, 0.20)**	1.80 (1.60, 2.00)**	1.30 (1.30, 1.30)*	0.30 (0.10, 6.00)	3.50 (3.30, 3.60)
Salt (g)	0.10 (0.01, 0.21)	0.10 (0.03, 0.35)	0.13 (0.08, 0.14)	0.07 (0.06, 0.07)*	0.10 (0.08, 0.10)	0.10 (0.10, 0.20)	0.11 (0.10, 0.11)	0.12 (0.10, 0.16)	0.10 (0.10, 0.20)
*Selected nutrients (non-mandatory to declare)*									
Fibre (g)^c^	0.80 (0.50, 1.30)***	0.60 (0.50, 1.30)**	0.35 (0.20, 0.50)*	0.00	0.00 (0.00, 0.40)	N/A	1.10 (0.10, 1.10)*	0.10 (0.00, 1.50)	0.00 (0.00, 0.50)
Vitamin D (mcg)^d^	1.10 (0.75, 1.10)**	0.75 (0.75, 0.75)	0.75 (0.75, 0.75)	0.75	0.75 (0.75, 0.75)	1.00 (1.00, 1.00)	0.75 (0.75, 0.75)	0.75 (0.75, 0.75)	1.00 (0.00, 1.00)
Vitamin B2 (mg)^e^	0.21 (0.20, 0.21)***	0.21 (0.21, 0.21)***	0.21 (0.21, 0.21)**	N/A	N/A	0.21 (0.21, 0.21)**	0.21 (0.21, 0.21)*	0.21 (0.21, 0.21)**	0.15 (0.14, 0.16)
Vitamin B12 (mcg)^f^	0.38 (0.38, 0.40)**	0.38 (0.30, 0.60)**	0.38 (0.38, 0.38)	0.38	0.38 (0.38, 0.38)	0.38 (0.38, 0.38)*	0.38 (0.38, 0.38)	0.38 (0.38, 0.38)*	0.58 (0.10, 0.59)
Calcium (mg)^g^	120 (120, 120)	120 (12, 120)	120 (10, 120)	120	120 (120, 120)	120 (120, 120)	120 (120, 120)	120 (10, 120)	120 (74, 124)
Iodine (mcg)^h^	22.50 (16.00, 23.00)**	N/A	N/A	N/A	N/A	N/A	N/A	N/A	12.00 (6.00, 25.80)

Significant difference between PBMAs and milk: * P < 0.05, **P < 0.01, ***P < 0.001.

N/A: data not available.

a Data for cashew nut-based (n = 1) and broad bean-based (n = 1) products are presented in Appendix A.

b Mixed-based products were soybean and pea, soybean and coconut, coconut and almond, and coconut and rice.

c Number of plant-based products reporting dietary fibre content: oat-based (n = 17), soy-based (n = 12), almond-based (n = 6), coconut-based (n = 1), rice-based (n = 4), potato-based (n = 3), mixed-based (n = 8).

d Number of plant-based products reporting vitamin D content: oat-based (n = 22), soy-based (n = 11), almond-based (n = 4), coconut-based (n = 1), rice-based (n = 4), pea-based (n = 5), potato-based (n = 3), mixed-based (n = 7).

e Number of plant-based products reporting vitamin B2 content: oat-based (n = 19), soy-based (n = 11), almond-based (n = 4), pea-based (n = 5), potato-based (n = 3), mixed-based (n = 4).

f Number of plant-based products reporting vitamin B12 content: oat-based (n = 19), soy-based (n = 9), almond-based (n = 4), coconut-based (n = 1), rice-based (n = 4), pea-based (n = 5), potato-based (n = 3), mixed-based (n = 6).

g Number of plant-based products reporting calcium content: oat-based (n = 19), soy-based (n = 12), almond-based (n = 6), coconut-based (n = 1), rice-based (n = 4), pea-based (n = 5), potato-based (n = 3), mixed-based (n = 9).

h Number of plant-based products reporting iodine content: oat-based (n = 14).

According to Table 3, the median energy content of almond- and coconut-based milk alternatives was lower than that of milk (medians: ≤18 vs 50 kcal/100 g, P_value_ <0.05). Although the median fat content of all ingredient subgroups of PBMAs was not different from milk, the saturated fat was generally lower in most PBMAs, particularly products based on oats, soybeans, almonds, and rice (medians: ≤0.30 vs 1.00 g/100 g, P_value_ <0.05). The median total sugar content of soy-, almond-, coconut-, pea-, potato-, and mixed-based milk alternatives was also lower than milk (medians: ≤2.50 vs 4.90 g/100 g, P_value_ <0.05). Similarly, the median protein content of most PBMAs, including oat-, almond-, coconut-, rice-, pea- and potato-based milk alternatives, was lower than milk (medians: ≤1.80 vs 3.50 g/100 g, P_value_ <0.05). The median protein content of soy-based milk alternatives was similar to milk (3.30 vs 3.50 g/100 g, P_value_ >0.05). The median salt content of coconut-based milk alternatives was also lower than milk (median: 0.07 vs 0.10 g/100 g, P_value_ <0.05), and the median fibre content of oat-, soy-, almond- and potato-based milk alternatives was higher than milk (medians: ≥0.35 vs 0 g/100 g, P_value_ <0.05).

Regarding products that declared micronutrients, oat-based milk alternatives had higher median vitamin D content (1.10 vs 1.00 mcg/100 g, P_value_ <0.01), and all subgroups of PBMAs had higher median vitamin B2 content (0.21 vs 0.15 mg/100 g, P_value_ <0.05) compared to milk. The median vitamin B12 content of oat-, soy- and mixed-based milk alternatives was lower than milk (0.38 vs 0.58 mcg/100 g, P_value_ <0.05). The median calcium content of all subgroups of PBMAs was similar to milk (120 mg/100 g), and the median iodine content of oat-based milk alternatives was higher than milk (22.50 vs 12.00 mcg/100 g, P_value_ <0.01).

PBYAs were mainly based on oats, soybeans, almonds and coconut, and some products contained a mixture of oats and peas; oats and potatoes; oats, coconut, and broad beans; and almonds and potatoes. Table 4 provides information on the energy and nutrient contents of PBYAs according to the main ingredient compared to yoghurt. Coconut-based yoghurt alternatives had higher median energy, fat, saturated fat, and lower median total sugar content than yoghurt (P_value_ <0.05). The median saturated fat content of oat-, soy- and almond-based yoghurt alternatives was lower than yoghurt (medians: ≤0.30 vs 1.65 g/100 g, P_value_ <0.05). The median protein content of all subgroups of PBYAs was lower than yoghurt (medians: ≤1.50 vs 3.45 g/100 g, P_value_ <0.05), except for soy-based products (3.80 vs 3.45 g/100 g, P_value_ <0.01). The median salt content of the oat- and soy-based yoghurt alternatives was lower and higher than yoghurt, respectively (P_value_ <0.05). In terms of products that declared fibre and micronutrients, the median fibre, vitamins D, B2, and B12 and calcium content of all subgroups of PBYAs (except coconut-based alternatives) was higher than yoghurt (P_value_ <0.05). The median iodine content in oat- and mixed-based yoghurt alternatives was also higher than in yoghurt (22.50 vs 10.15 mcg/100 g, P_value_ <0.05).

**Table 4:** Median (min., max.) of energy and nutrients in 100 g PBYAs (Plant-based Yoghurt Alternatives) according to main yoghurt substitute ingredient and comparison with yoghurt.

	Oat (n = 4)	Soybean (n = 21)	Almond (n = 3)	Coconut (n = 5)	Mixed^a^ (n = 8)	Yoghurt (n = 20)
*Energy and nutrients (mandatory to declare)*						
Energy (kcal)	79 (70, 86)	72 (42, 120)	66 (59, 67)	110 (84, 179)*	81 (41, 145)	64 (39, 129)
Total fat (g)	2.15 (1.90, 2.50)	2.20 (1.80, 10.00)	1.90 (1.90, 1.90)	7.70 (5.60, 18.00)**	2.60 (0.90, 11.00)	2.60 (0.05,8.30)
Saturated fat (g)	0.20 (0.20, 0.20)*	0.30 (0.00, 5.80)**	0.20 (0.10, 0.20)*	7.10 (5.10, 16.00)**	0.25 (0.00, 2.50)	1.65 (0.00,5.30)
Carbohydrate (g)	14.00 (9.20, 15.10)	7.90 (0.00, 12.00)	10.00 (9.30, 10.00)	6.00 (4.20, 13.00)	13.00 (4.20, 16.00)	9.35 (4.20,15.00)
Total sugar (g)	8.15 (4.80, 8.30)	7.40 (0.00, 10.10)	7.90 (6.00, 8.00)	1.80 (0.60, 9.10)*	6.15 (0.40, 9.50)	7.75 (2.90,14.50)
Protein (g)	1.00 (0.90, 1.10)**	3.80 (3.30, 5.80)**	0.90 (0.80, 0.90)**	1.00 (0.60, 1.40)***	1.50 (1.00, 3.30)***	3.45 (2.50,7.00)
Salt (g)	0.08 (0.06, 0.11)	0.14 (0.07, 0.36)***	0.10 (0.10, 0.10)	0.07 (0.03, 0.20)	0.10 (0.08, 0.11)	0.10 (0.00,0.10)
*Selected nutrients (non-mandatory to declare)*						
Fibre (g)^b^	0.90 (0.90, 0.90)**	1.05 (0.80, 2.60)***	0.20 (0.20, 0.20)*	0.00	0.90 (0.10, 0.90)**	0.00 (0.00,0.50)
Vitamin D (mcg)^c^	1.30 (1.10, 1.50)**	0.75 (0.60, 1.50)**	1.50 (1.50, 1.50)**	N/A	1.00 (1.00, 1.50)**	0.10 (0.00,1.00)
Vitamin B2 (mg)^d^	0.21 (0.21, 0.21)**	0.21 (0.21, 0.21)***	0.21 (0.21, 0.21)**	N/A	0.21 (0.21, 0.21)***	0.16 (0.14,0.32)
Vitamin B12 (mcg)^e^	0.49 (0.38, 0.60)**	0.38 (0.38, 0.38)***	0.38 (0.38, 0.38)*	N/A	0.40 (0.38, 0.50)**	0.26 (0.15,0.56)
Calcium (mg)^f^	120 (120, 120)*	120 (14, 120)**	120 (120, 120)	N/A	120 (120, 131)**	109.00 (20,136)
Iodine (mcg)^g^	22.50 (22.50, 22.50)*	N/A	N/A	N/A	22.50 (22.50, 25.00)***	10.15 (3.00,15.60)

Significant difference between PBYAs and yoghurt: *P < 0.05, **P < 0.01, ***P < 0.001.

N/A: data not available.

a Mixed-based products were oat and pea protein; oat and potato protein; oat, coconut and broad bean protein; almond and potato protein.

b Number of plant-based products reporting dietary fibre content: oat-based (n = 2), soy-based (n = 16), almond-based (n = 3), coconut-based (n = 1), mixed-based (n = 3).

c Number of plant-based products reporting vitamin D content: oat-based (n = 4), soy-based (n = 19), almond-based (n = 3), mixed-based (n = 8).

d Number of plant-based products reporting vitamin B2 content: oat-based (n = 4), soy-based (n = 9), almond-based (n = 3), mixed-based (n = 7).

e Number of plant-based products reporting vitamin B12 content: oat-based (n = 4), soy-based (n = 15), almond-based (n = 3), mixed-based (n = 8).

f Number of plant-based products reporting calcium content: oat-based (n = 4), soy-based (n = 16), almond-based (n = 3), mixed-based (n = 8).

g Number of plant-based products reporting iodine content: oat-based (n = 2), mixed-based (n = 6).

### 3.4 Nutritional profile of organic vs non-organic and plain vs flavoured products

The difference between organic and non-organic, as well as plain and flavoured products in PBMAs and PBYAs, are presented in Table 5, Table 6. Regarding organic and non-organic PBMAs, the main difference was in the vitamin and mineral content, as the median vitamin D content of organic PBMAs was higher (1.10 vs 0.75 mcg/100 g, P_value_ <0.05) and median calcium content was lower (10 vs 120 g/100 g, P_value_ <0.001) compared to non-organic PBMAs. None of the organic PBMAs declared their vitamins B2 and B12 or iodine content. Similar findings were observed for the difference between organic and non-organic PBYAs, except for vitamin D, where there was no statistically significant difference between organic and non-organic PBYAs (P_value_ >0.05). In terms of comparison between plain and flavoured PBMAs, the median energy, carbohydrate, total sugar, protein, salt, and fibre contents of flavoured products were higher than that of plain varieties (P_value_ <0.05). The median energy, carbohydrate, and total sugar contents of flavoured PBYAs were higher (P_value_ <0.05), and median fat and saturated fat contents were lower than plain PBYAs (P_value_ <0.05).

**Table 5:** Median (min., max.) of energy and nutrients in 100 g plant-based organic and non-organic PBMAs (Plant-based Milk Alternatives) and PBYAs (Plant-based Yoghurt Alternatives).

	PBMAs		PBYAs	
	Organic PBMAs (n = 11)	Non-organic PBMAs (n = 62)	Organic PBYAs (n = 10)	Non-organic PBYAs (n = 31)
*Energy and nutrients (mandatory to declare)*				
Energy (kcal)	38 (15, 50)	46 (13, 74)	85 (42, 179)	75 (41, 145)
Total fat (g)	1.40 (0.50, 2.30)	1.55 (0.50, 3.00)	3.95 (1.90, 18.00)	2.20 (0.90, 11.00)
Saturated fat (g)	0.30 (0.10, 0.80)	0.30 (0.00, 1.40)	2.75 (0.10, 16.00)	0.30 (0.00, 5.70)
Carbohydrate (g)	2.30 (0.00, 9.90)	4.90 (0.00, 12.00)	5.80 (0.00, 13.00)	9.30 (0.00, 16.00)
Total sugar (g)	1.90 (0.00, 7.10)	2.85 (0.00, 11.00)	3.00 (0.00, 9.10)	7.80 (0.00, 10.10)
Protein (g)	1.00 (0.10, 4.00)	1.20 (0.00, 6.00)	3.40 (0.90, 4.10)	3.30 (0.60, 5.80)
Salt (g)	0.10 (0.03, 0.14)	0.10 (0.01, 0.35)	0.13 (0.03, 0.20)	0.10 (0.06, 0.36)
*Selected nutrients (non-mandatory to declare)*				
Fibre (g)^a^	0.55 (0.00, 0.80)	0.60 (0.00, 1.50)	0.90	0.90 (0.00, 2.60)
Vitamin D (mcg)^b^	1.10 (1.10, 1.10)	0.75 (0.75, 1.50)*	0.85 (0.85, 1.00)	1.00 (0.60, 1.50)
Vitamin B2 (mg)^c^	N/A	0.21 (0.20, 0.21)	N/A	0.21 (0.21, 0.21)
Vitamin B12 (mcg)^d^	N/A	0.38 (0.38, 0.60)	N/A	0.38 (0.30, 0.60)
Calcium (mg)^e^	10 (10, 12)	120 (120, 120)***	14.00	120 (108, 131)**
Iodine (mcg)^f^	N/A	22.50 (16.00, 23.00)	N/A	22.50 (22.50, 25.00)

Significant difference between organic and non-organic products: *P < 0.05, **P < 0.01, ***P < 0.001.

N/A: data not available.

a Number of plant-based products reporting dietary fibre content: organic PBMAs (n = 8), non-organic PBMAs (n = 44), organic PBYAs (n = 1), non-organic PBYAs (n = 24).

b Number of plant-based products reporting vitamin D content: organic PBMAs (n = 3), non-organic PBMAs (n = 56), organic PBYAs (n = 4), non-organic PBYAs (n = 30).

c Number of plant-based products reporting vitamin B2 content: non-organic PBMAs (n = 48), non-organic PBYAs (n = 23).

d Number of plant-based products reporting vitamin B12 content: non-organic PBMAs (n = 53), non-organic PBYAs (n = 30).

e Number of plant-based products reporting calcium content: organic PBMAs (n = 3), non-organic PBMAs (n = 58), organic PBYAs (n = 1), non-organic PBYAs (n = 30).

f Number of plant-based products reporting iodine content: non-organic PBMAs (n = 14), non-organic PBYAs (n = 8).

**Table 6:** Median (min., max.) of energy and nutrients in 100 g plain and flavoured PBMAs (plant-based milk alternatives) and PBYAs (plant-based yoghurt alternatives).

	PBMAs		PBYAs	
	Plain PBMAs (n = 59)	Flavoured PBMAs (n = 14)	Plain PBYAs (n = 15)	Flavoured PBYAs (n = 26)
*Energy and nutrients (mandatory to declare)*				
Energy (kcal)	40 (13, 74)	60 (35, 74)***	68 (41, 179)	76 (59, 152)**
Total fat (g)	1.50 (0.50, 3.00)	2.25 (0.50, 3.00)	2.50 (0.90, 18.00)	1.95 (1.00, 14.00)**
Saturated fat (g)	0.30 (0.00, 1.40)	0.30 (0.10, 0.60)	0.40 (0.00, 16.00)	0.25 (0.00, 12.00)*
Carbohydrate (g)	2.70 (0.00, 12.00)	6.40 (3.00, 11.00)**	4.30 (0.00, 12.00)	10.00 (5.80, 16.00)***
Total sugar (g)	2.50 (0.00, 11.00)	5.00 (3.00, 8.90)***	2.20 (0.00, 4.80)	8.00 (3.60, 10.10)***
Protein (g)	1.00 (0.00, 5.00)	1.45 (1.00, 6.00)**	3.30 (0.60, 5.80)	3.50 (0.80, 5.10)
Salt (g)	0.10 (0.03, 0.35)	0.12 (0.01, 0.20)*	0.10 (0.03, 0.36)	0.11 (0.03, 0.31)
*Selected nutrients (non-mandatory to declare)*				
Fibre (g)^a^	0.50 (0.00, 1.30)	1.10 (0.50, 1.50)**	0.90 (0.00, 2.60)	0.90 (0.20, 2.40)
Vitamin D (mcg)^b^	0.75 (0.75, 1.50)	1.00 (0.75, 1.10)	1.05 (0.75, 1.50)	1.00 (0.60, 1.50)
Vitamin B2 (mg)^c^	0.21 (0.20, 0.21)	0.21 (0.21, 0.21)	0.21 (0.21, 0.21)	0.21 (0.21, 0.21)*
Vitamin B12 (mcg)^d^	0.38 (0.38, 0.60)	0.38 (0.38, 0.38)	0.38 (0.38, 0.60)	0.38 (0.30, 0.60)
Calcium (mg)^e^	120 (10, 120)	120 (120, 120)	120 (14, 131)	120 (108, 120)
Iodine (mcg)^f^	22.50 (16.00, 23.00)	22.50 (22.50, 22.50)	23.75 (22.50, 25.00)	22.50 (22.50, 22.50)

Significant difference between plain and flavoured products: *P < 0.05, **P < 0.01, ***P < 0.001.

N/A: data not available.

a Number of plant-based products reporting dietary fibre content: plain PBMAs (n = 41), flavoured PBMAs (n = 11), plain PBYAs (n = 9), flavoured PBYAs (n = 16).

b Number of plant-based products reporting vitamin D content: plain PBMAs (n = 46), flavoured PBMAs (n = 13), plain PBYAs (n = 10), flavoured PBYAs (n = 24).

c Number of plant-based products reporting vitamin B2 content: plain PBMAs (n = 35), flavoured PBMAs (n = 13), plain PBYAs (n = 5), flavoured PBYAs (n = 18).

d Number of plant-based products reporting vitamin B12 content: plain PBMAs (n = 43), flavoured PBMAs (n = 10), plain PBYAs (n = 9), flavoured PBYAs (n = 21).

e Number of plant-based products reporting calcium content: plain PBMAs (n = 48), flavoured PBMAs (n = 13), plain PBYAs (n = 10), flavoured PBYAs (n = 21).

f Number of plant-based products reporting iodine content: plain PBMAs (n = 5), flavoured PBMAs (n = 5), plain PBYAs (n = 2), flavoured PBYAs (n = 6).

### 3.5 Nutritional profile of plant-based cheese alternatives

The energy and nutrient contents of PBChAs are presented in Table 7. The median saturated fat in plant fat-based cheese alternatives was higher than in cheese (21.00 vs 14.45 g/100 g, P_value_ <0.001). The median carbohydrate and salt contents of both subgroups of PBChAs were higher, and the median protein was lower than cheese (P_value_ <0.05). Regarding those products that declared fibre and micronutrients, the median fibre, vitamins D and B2, calcium, and iodine contents in plant fat- and protein-based cheese alternatives were higher than in cheese (P_value_ <0.05). The median vitamin B12 content in both subgroups of PBChAs was higher than in cheese (medians: ≥1.70 vs 0.62 mcg/100 g, P_value_ <0.05).

**Table 7:** Median (min., max.) of energy and nutrients in 100 g PBChAs (Plant-based Cheese Alternatives) and comparison with cheese.

	Plant fat-based cheese (n = 21)	Plant fat- and protein-based cheese (n = 15)^a^	Cheese (n = 36)
*Energy and nutrients (mandatory to declare)*			
Energy (kcal)	285 (213, 337)	292 (222, 628)	283 (100, 613)
Total fat (g)	23.00 (20.00, 29.00)	24.00 (13.00, 68.00)	22.25 (4.00, 65.14)
Saturated fat (g)	21.00 (18.00, 26.00)***	14.00 (2.50, 23.00)	14.45 (2.60, 27.90)
Carbohydrate (g)	22.00 (8.00, 32.00)***	19.00 (3.30, 30.00)***	1.90 (0.00, 8.20)
Total sugar (g)	0.50 (0.00, 0.80)	0.30 (0.00, 3.60)	0.20 (0.00, 5.40)
Protein (g)	0.50 (0.00, 1.00)***	1.10 (0.00, 7.00)***	17.05 (4.00, 36.20)
Salt (g)	1.80 (1.20, 3.00)**	2.20 (0.71, 3.50)*	1.20 (0.10, 4.20)
*Selected nutrients (non-mandatory to declare)*			
Fibre (g)^b^	N/A	6.10 (0.80, 6.10)***	0.00 (0.00, 0.00)
Vitamin D (mcg)^c^	N/A	4.00 (4.00, 4.00)**	0.12 (0.00, 0.68)
Vitamin B2 (mg)^d^	N/A	0.90 (0.90, 0.90)**	0.27 (0.10, 0.55)
Vitamin B12 (mcg)^e^	2.50 (1.50, 2.50)***	1.70 (0.50, 2.50)**	0.62 (0.13, 3.30)
Calcium (mg)^f^	650 (650, 650)	135 (120, 150)*	440 (50, 1025)
Iodine (mcg)^g^^,^^h^	N/A	87.00 (87.00, 87.00)**	12.00 (0.50, 72.00)

Significant difference between PBChAs and cheese: *P < 0.05, **P < 0.01, ***P < 0.001.

N/A: Data not available.

a Protein-containing ingredients include nuts, seeds, or protein isolates, e.g., potato protein.

b Number of plant-based products reporting dietary fibre content: plant fat- and protein-based cheese (n = 3).

c Number of plant-based products reporting vitamin D content: plant fat- and protein-based cheese (n = 4).

d Number of plant-based products reporting vitamin B2 content: plant fat- and protein-based cheese (n = 3).

e Number of plant-based products reporting vitamin B12 content: plant fat-based cheese (n = 19), plant fat- and protein-based cheese (n = 11).

f Number of plant-based products reporting calcium content: plant fat-based cheese (n = 6), plant fat- and protein-based cheese (n = 4).

g Number of plant-based products reporting iodine content: plant fat- and protein-based cheese (n = 4).

h In the Swedish Food Composition Database, 31 cheese items had iodine values.

### 3.6 Nutritional profile of plant-based cream, ice cream, and fat-spread alternatives

The energy and nutrient contents of PBCrAs and PBIAs are presented in Table 8, Table 9. The median total sugar content in plant fat- and protein-based cream alternatives was lower than in cream (2.35 vs 3.50 g/100 g, P_value_ <0.05). The median protein content in plant fat-based cream alternatives was lower compared to cream (0.95 vs 2.95 g/100 g, P_value_ <0.001), and median fibre content in both subgroups of PBCrAs was higher than in cream (medians ≥0.5 vs 0 g/100 g, P_value_ <0.01). In PBIAs, the median energy, total sugar, and fibre contents were higher, and the median protein content was lower than ice cream (P_value_ <0.01).

**Table 8:** Median (min., max.) of energy and nutrients in 100 g PBCrAs (Plant-based Cream Alternatives) and comparison with cream.

	Plant fat-based cream (n = 16)	Plant fat- and protein-based cream (n = 6)^a^	Cream (n = 10)
*Energy and nutrients (mandatory to declare)*			
Energy (kcal)	169 (80, 420)	167 (137, 268)	162 (84, 374)
Total fat (g)	15.00 (7.00, 28.00)	15.00 (12.00, 24.60)	15.00 (5.16, 40.00)
Saturated fat (g)	7.45 (0.90, 26.00)	9.85 (1.60, 18.60)	9.70 (3.30, 25.60)
Carbohydrate (g)	6.90 (0.00, 47.00)	4.30 (1.20, 9.50)	4.35 (3.00, 6.70)
Total sugar (g)	3.60 (0.00, 44.00)	2.35 (1.20, 3.60)*	3.50 (2.60, 4.30)
Protein (g)	0.95 (0.00, 3.00)***	2.20 (1.00, 3.50)	2.95 (2.00, 3.80)
Salt (g)	0.11 (0.01, 0.34)	0.10 (0.06, 1.20)	0.10 (0.10, 1.20)
*Selected nutrients (non-mandatory to declare)*			
Fibre (g)^b^	0.60 (0.50, 1.00)***	0.50 (0.40, 0.60)**	0.00 (0.00, 0.00)
Vitamin D (mcg)^c^	N/A	1.00	0.10 (0.03, 0.24)
Vitamin B2 (mg)^d^	N/A	0.21	0.13 (0.09, 0.17)
Vitamin B12 (mcg)^e^	N/A	0.40	0.29 (0.10, 0.52)
Calcium (mg)^f^	69 (18, 120)	11 (10, 120)	95 (52, 114)
Iodine (mcg)^g^	N/A	22.50	8.35 (4.00, 10.70)

Significant difference between PBCrAs and cream: *P < 0.05, **P < 0.01, ***P < 0.001.

N/A: Data not available.

a Protein-containing ingredients include soybean or protein isolates, e.g., broad bean protein.

b Number of plant-based products reporting dietary fibre content: plant fat-based cream (n = 7), plant fat- and protein-based cream (n = 2).

c Number of plant-based products reporting vitamin D content: plant fat- and protein-based cream (n = 1).

d Number of plant-based products reporting vitamin B2 content: plant fat- and protein-based cream (n = 1).

e Number of plant-based products reporting vitamin B12 content: plant fat- and protein-based cream (n = 1).

f Number of plant-based products reporting calcium content: plant fat- and protein-based cream (n = 2), plant protein-based cream (n = 3).

g Number of plant-based products reporting iodine content: plant fat and protein-based cream (n = 1).

**Table 9:** Median (min., max.) of energy and nutrients in 100 g PBIAs (Plant-based Ice cream Alternatives) and comparison with ice cream.

	PBIAs (n = 23)	Ice cream (n = 12)
*Energy and nutrients (mandatory to declare)*		
Energy (kcal)	232 (169, 276)**	195 (139, 348)
Total fat (g)	11.00 (4.50, 15.00)	9.44 (4.70, 25.33)
Saturated fat (g)	6.60 (1.20, 10.50)	5.45 (2.00, 12.90)
Carbohydrate (g)	31.00 (24.00, 37.60)***	22.85 (9.20, 27.00)
Total sugar (g)	22.00 (13.90, 28.00)***	17.10 (6.30, 21.60)
Protein (g)	1.60 (0.50, 3.60)***	3.50 (2.40, 4.60)
Salt (g)	0.14 (0.00, 0.35)	0.10 (0.00, 0.50)
*Selected nutrients (non-mandatory to declare)*		
Fibre (g)^a^	1.10 (0.70, 1.40)**	0.00 (0.00, 1.20)
Vitamin D (mcg)	N/A	0.05 (0.00, 0.66)
Vitamin B2 (mg)	N/A	0.17 (0.10, 0.33)
Vitamin B12 (mcg)	N/A	0.46 (0.17, 0.70)
Calcium (mg)	N/A	116 (55, 141)
Iodine (mcg)^b^	N/A	9.85 (3.90, 18.60)

Significant difference between PBIAs and ice cream: **P < 0.01, ***P < 0.001.

N/A: Data not available.

a Number of plant-based products reporting dietary fibre content: PBIAs (n = 5).

b In the Swedish Food Composition Database, 10 ice cream items had iodine values.

The nutrient profile of PBSAs is shown in Table 10. The median energy content for PBSAs was lower than butter (681 vs 728 kcal/100 g, P_value_ <0.05) and higher than blended margarine (681 vs 535 kcal/100 g, P_value_ <0.05). The median total fat content of PBSAs was lower than butter (75.00 vs 81.80 g/100 g, P_value_ <0.05), and similarly, their saturated fat content was also lower than butter (20.00 vs 52.40 g/100 g, P_value_ <0.01). In PBSAs, median monounsaturated fat was higher than in butter (32.00 vs 22.70 g/100 g, P_value_ <0.05), and median polyunsaturated fat was higher than in butter and blended margarine (15.00 vs ≤ 8.40 g/100 g, P_value_ <0.01). The PBSAs’ median salt content was lower than blended margarine (1.00 vs 1.30 g/100 g, P_value_ <0.01). In PBSAs that declared micronutrients, the median vitamin D content was higher than butter (20.00 vs 0.56 mcg/100 g, P_value_ <0.01), the median vitamin B2 content was higher than blended margarine (1.40 vs 0 mg/100 g, P_value_ <0.01), and median vitamin B12 content was higher than butter and blended margarine (2.50 vs 0 g/100 mcg, P_value_ <0.05).

**Table 10:** Median (min., max.) of energy and nutrients in 100 g PBSAs (Plant-based fat Spread Alternatives) and comparison with butter and blended animal- and plant-based margarine.

	PBSAs (n = 27)	Butter (n = 3)	Blended margarine (n = 13)
*Energy and nutrients (mandatory to declare)*			
Energy (kcal)	681 (315, 900)	728 (728, 729)*	535 (272, 712)*
Total fat (g)	75.00 (35.00, 100.00)	81.80 (81.80, 82.00)*	60.00 (28.00, 80.00)
Saturated fat (g)	20.00 (6.80, 91.00)	52.40 (52.40, 55.00)**	18.00 (11.20, 39.20)
Carbohydrate (g)	0.00 (0.00, 4.00)	0.50 (0.50, 0.50)	0.50 (0.00, 6.20)*
Total sugar (g)	0.00 (0.00, 2.90)	0.40 (0.40, 0.50)	0.40 (0.20, 0.80)
Protein (g)	0.00 (0.00, 0.50)	0.60 (0.40, 0.60)*	0.50 (0.20, 2.00)**
Salt (g)	1.00 (0.00, 1.70)	1.20 (0.00, 2.00)	1.30 (1.00, 2.00)**
*Selected nutrients (non-mandatory to declare)*			
Monounsaturated fat (g)^a^	32.00 (11.00, 48.00)	22.70 (19.10, 22.70)*	26.50 (12.00, 44.30)
Polyunsaturated fat (g)^b^	15.00 (7.00, 41.00)	2.90 (2.90, 3.00)**	8.40 (4.00, 20.90)**
Fibre (g)^c^	0.00	0.00 (0.00, 0.00)	0.00 (0.00, 0.00)
Vitamin D (mcg)^d^	20.00 (6.80, 20.00)	0.56 (0.51, 0.56)**	20.00 (7.50, 20.00)
Vitamin B2 (mg)^e^	1.40 (1.40, 1.40)	0.02 (0.00, 0.02)	0.00 (0.00, 0.09)**
Vitamin B12 (mcg)^f^	2.50 (2.50, 2.50)	0.00 (0.00, 0.00)*	0.00 (0.00, 0.10)**
Calcium (mg)	N/A	15.00 (0.00, 18.00)	0.00 (0.00, 60.00)
Iodine (mcg)^g^	N/A	4.00	0.45 (0.00, 2.90)

Significant difference between PBSAs and animal-based fat spread (butter/blended margarine): *P < 0.05, **P < 0.01.

N/A: data not available.

a Number of plant-based products reporting monounsaturated fat content: PBSAs (n = 21).

b Number of plant-based products reporting polyunsaturated fat content: PBSAs (n = 21).

c Number of plant-based products reporting dietary fibre content: PBSAs (n = 1).

d Number of plant-based products reporting vitamin D content: PBSAs (n = 22).

e Number of plant-based products reporting vitamin B2 content: PBSAs (n = 2).

f Number of plant-based products reporting vitamin B12 content: PBSAs (n = 2).

g In the Swedish Food Composition Database, 1 butter item and 10 blended margarine items had iodine values.

## 4 Discussion

This is the first study investigating the nutritional properties of plant-based dairy alternatives in the Swedish market. The interest in plant-based foods is increasing, and manufacturers address this demand by producing innovative products that resemble animal-based foods. Therefore, assessing the nutritional properties of plant-based foods, such as alternatives to dairy products, is important to shed light on their similarities and differences when compared with animal-based foods.

The findings of this study revealed that PBMAs and PBYAs had some advantages over dairy products, such as lower saturated fat and higher fibre content. However, they also had some disadvantages, including lower protein content than dairy products. The lack of micronutrient fortification in some products was another limitation of these plant-based dairy alternatives. Organic PBMAs and PBYAs appeared nutritionally inferior to non-organic varieties due to the lack of fortification for most micronutrients (except vitamin D). Flavoured PBMAs and PBYAs had higher energy and total sugar content compared to plain products. PBChAs also had lower protein and higher fibre content than cheese. Regarding PBCrAs, PBIAs and PBSAs, they do not appear to be nutritionally different from corresponding dairy products, except for instances such as the lower saturated fat content of PBSAs compared to butter. Fortification was most common in PBMAs, BPYAs and to some extent in PBChAs, while PBCrAs, PBIAs and PBSAs were rarely fortified with several micronutrients.

The energy range of PBMAs in the Swedish market was similar to or lower than milk. Thus, replacing milk with PBMAs may not increase energy intake. However, apart from PBMAs, the energy content of other plant-based dairy alternatives was not necessarily lower than corresponding dairy products. Therefore, the lower energy content of plant-based products compared to animal-based products is not generalisable to all plant-based foods. Moreover, this finding is not generalisable to other markets. For example, in a U.S. study, the energy content of PBMAs varied and, in some cases, exceeded the energy content of milk (Chalupa-Krebzdak et al., 2018).

The total fat content of most plant-based dairy alternatives was similar to corresponding dairy products, but their saturated fat content was lower than dairy except for coconut-based products (e.g., coconut-based yoghurt alternatives). Therefore, replacing dairy with these plant-based alternatives can reduce the dietary intake of saturated fat. The reduction in saturated fat intake is associated with a decrease in the incidence of cardiovascular disease (Sacks et al., 2017). It is worth mentioning that although dairy products contain saturated fat, no association between dairy intake and cardiovascular disease was observed. On the contrary, the intake of some dairy products, such as cheese, was associated with a lower risk of this disease (Guo et al., 2017). It has been suggested that although dairy products contain saturated fat, they contain other nutrients that prevent cardiovascular events; therefore, the combination of all nutrients in a product affects the health indicators (Feeney and McKinley, 2020).

Protein is one of the key nutrients in dairy products. Among all plant-based dairy alternatives, only the quantity of protein in soy-based products was similar to that of dairy. Thus, replacing dairy with plant-based alternatives can reduce protein intake, which may have a negative health impact on vulnerable populations such as children (Morency et al., 2017). In terms of protein quality, while whey protein digestibility is 100%, the digestibility of plant-based proteins such as soy protein is between 72% and 86%, depending on the food processing (Boye et al., 2012). Plant-based protein also lacks some essential amino acids; for example, soy, pea and almond proteins lack methionine and cysteine (Boye et al., 2012). Hence, other foods in the diet should complement the missing amino acids. It also might be possible to use a combination of multiple plant protein sources to compensate for missing essential amino acids. In this Swedish study, it was impossible to investigate the amino acid composition of the plant-based dairy alternatives; however, most products that used a mixture of protein sources had low protein quantity. Therefore, both protein quality and quantity should be considered when assessing plant-based dairy alternatives’ nutritional quality.

The findings of this Swedish study regarding the low protein content of these products are similar to other studies in the U.S. (Craig et al., 2022; Drewnowski, 2022), UK (Clegg et al., 2021), Australia (Zhang et al., 2020), Spain (Fresán and Rippin, 2021), and Norway (Tonheim et al., 2022). However, the quantity of protein varies between different markets. For example, the protein content of most PBYAs in the U.S. market was higher than in the Swedish market (Craig and Brothers, 2021). The protein content of nut-based and soy-based cheeses in Spain was also higher (Fresán and Rippin, 2021) than in this Swedish study. Similar to other studies (Craig and Brothers, 2021; Drewnowski, 2022; Fresán and Rippin, 2021), the protein quantity of soy-based dairy alternatives was comparable to dairy products. However, some studies in the U.S., Australia, and Europe demonstrated that not only soy-based but also pea-based milk and yoghurt alternatives had a similar protein content to milk and yoghurt (Craig and Brothers, 2021; Craig and Fresán, 2021). In this Swedish study, only soy-based products had high protein content. This discrepancy in protein content could be due to differences in product formulations, processing methods, and the quantity of the main ingredient in the product.

In this Swedish study, most PBMAs, PBYAs, and PBSAs were fortified with vitamin D. According to the Swedish Food Agency, plain PBMAs and flavoured and plain PBYAs with a maximum fat content of 3%, as well as PBSA (i.e. margarine), should contain a certain amount of vitamin D (Swedish Food Agency, 2019); thus, these products should be fortified with vitamin D. The legislation is also applicable to corresponding milk, yoghurt, and blended margarine (Swedish Food Agency, 2019). It is worth noting that butter is not fortified, as it naturally contains vitamin D (Schmid and Walther, 2013). Furthermore, food fortification legislation stipulates that organic products should only be fortified with vitamin D (Swedish Food Agency, 2019); thus, these products might be nutritionally less suitable for vegans and vegetarians since they are not fortified with other micronutrients, such as vitamin B12 and calcium. Fortifying plant-based dairy alternatives with vitamin D and other vitamins and minerals can help vegans and vegetarians meet the required intake of these micronutrients (Craig and Fresán, 2021).

The majority of PBMAs and PBYAs were fortified with calcium; thus, their calcium content was similar to milk and yoghurt. However, the bioavailability of calcium in plant-based dairy alternatives may differ from that of dairy products. For example, lactose can increase the bioavailability of calcium, whereas other sugars do not have such an effect (Chalupa-Krebzdak et al., 2018; Miller, 1989). The absorption of calcium in fortified products depends on the type of calcium compound, its solubility, and the presence of antinutrients (Chalupa-Krebzdak et al., 2018). For instance, the bioavailability of calcium from tri-calcium phosphate is less than calcium carbonate (Chaiwanon et al., 2000), and the solubility of both of these calcium compounds is low in fortified products, which affects their ingestion (Heaney and Rafferty, 2006; Heaney et al., 2005). Antinutrients such as phytate in nuts, seeds, cereals, and pulses, in particular oats and broad beans, reduce calcium bioavailability (Dendougui and Schwedt, 2004; López-Moreno et al., 2022). Food processing techniques, including soaking, cooking, germination, and fermentation, can reduce the phytate content of foods (López-Moreno et al., 2022). Therefore, the bioavailability of calcium should be taken into consideration when comparing plant-based dairy alternatives with dairy.

Cream, ice cream, butter, and margarine are energy-dense and nutrient-poor foods (Rangan et al., 2009) and mostly have high fat and low calcium content (U.S. Department of Agriculture, 2023); thus, their role in the diet differs from milk, yoghurt, and cheese. Dietary guidelines recommend limiting the intake of energy-dense and nutrient-poor foods (Australian National Health and Medical Research Council, 2013, U.S. Department of Agriculture and U.S. Department of Health and Human Services, 2020). Therefore, the intake of products such as cream, ice cream, and fat spreads should be limited. This Swedish study demonstrated that the higher fibre content was the main advantage of PBCrAs and PBIAs over cream and ice cream. The beneficial feature of PBSAs over corresponding animal-based products was their healthier fat profile (lower saturated fat and higher mono- and polyunsaturated fats) and higher vitamin D and B12 content in fortified items. Since PBCrAs, PBIAs, and PBSAs are also energy-dense foods, their intake should be limited, and some of the mentioned characteristics, such as high fibre content, may be less important when the quantity of intake is low.

Milk contains some compounds, such as bioactive peptides, which have antibacterial activity in the gastrointestinal tract and act as an immune system stimulant (Kanekanian, 2014). One of the limitations of plant-based dairy products is the lack of these bioactive compounds, as well as the lower content of other nutrients, including choline, vitamin A, magnesium, and potassium, compared to milk (Cifelli et al., 2022). This limitation could affect vulnerable groups, such as infants, children, adolescents, and older adults (Scholz-Ahrens et al., 2020). In this Swedish study, information on the content of these nutrients was missing for the plant-based dairy alternatives. Therefore, it is important to consider other key nutrients and components when comparing plant-based dairy alternatives with dairy products.

Despite the mentioned limitations, these products have some nutrients and compounds with health benefits. For example, their fibre content is higher than dairy. Fibre, particularly soluble fibre such as beta-glucan in oats, has a beneficial impact on metabolic diseases, e.g. obesity, hypertension, dyslipidaemia, and insulin resistance (El Khoury et al., 2012). Antinutrients may also have health benefits. Some of the beneficial features of phytates include antioxidant and neuroprotective effects and insulin response improvement in patients with diabetes (López-Moreno et al., 2022). Since food processing may modify the molecular structure of fibres (Goudar et al., 2020) and reduce the antinutrient content (López-Moreno et al., 2022), this might also lessen the health benefits. Furthermore, the beneficial impact of fibres and other nutrients and compounds depends on the quantity of consumption.

This study has some strengths and limitations. It investigated the nutritional quality of plant-based alternatives to six different groups of dairy products. The data were collected from the market and manufacturers rather than from databases. Both online information and product labels were checked for inconsistencies to ensure accuracy. However, the possibility of discrepancies between online information and product labels due to a lack of updates cannot be ruled out because the formulation of plant-based dairy alternatives may change rapidly in the market.

One of the limitations of this study was the lack of information on various micronutrients, such as vitamin A and zinc, for a comprehensive assessment of these products. The complete nutritional profile of foods is usually obtained by laboratory analyses, but due to high costs, the number of products with a complete nutritional profile is limited. Furthermore, similar to other studies (Cifelli et al., 2022), this Swedish study could not quantitatively consider the bioavailability of key nutrients such as protein and calcium in plant-based products. Moreover, this study did not cover other important aspects of plant-based dairy alternatives, including their food safety and allergenicity properties. These features should be considered along with nutritional characteristics for product development and dietary recommendations.

## 5 Conclusion

The plant-based dairy alternatives on the Swedish market have nutritional strengths and weaknesses compared to corresponding dairy products. Our findings indicate that replacing dairy products with fortified plant-based alternatives can provide fibre and micronutrients. Furthermore, plant-based dairy alternatives may reduce saturated fat intake compared to milk, yoghurt and butter. However, replacing dairy products with plant-based alternatives risks compromising protein intake. Moreover, consuming organic PBMAs and PBYAs can decrease micronutrient intake and consuming the flavoured variety of these alternatives may increase energy and sugar intake. The findings of this study can support product development by highlighting the potential nutritional strengths and the need for improvement in some features.

The nutritional impact of replacing dairy products with plant-based alternatives depends on the quantity of intake and total or partial replacement of dairy in the diet. When dairy consumption is high, a high degree of replacement with plant-based dairy alternatives can increase fibre intake and lower the consumption of protein and saturated fat. Therefore, the nutritional benefits and drawbacks of plant-based dairy alternatives should be considered when planning to use these products as dairy substitutes, particularly in vulnerable groups.

## Ethical approval

Not Applicable.

## Funding

This study was part of the FINEST (Food Innovation Enabling Sustainable Transition) project. FINEST is supported by the 10.13039/501100001862Swedish Research Council for Environment, Agricultural Sciences and Spatial Planning (FORMAS) [Grant no. 2020-02839]. The funder and industrial partners had no role in the study design, data collection, analyses, interpretation, and manuscript preparation.

## CRediT authorship contribution statement

**Hanieh Moshtaghian:** Conceptualization, Methodology, Data curation, Formal analysis, Writing – original draft, and, Writing – review & editing. **Elinor Hallström:** Conceptualization, Methodology, Funding acquisition, Writing – review & editing. **Marta Bianchi:** Conceptualization, Writing – review & editing. **Susanne Bryngelsson:** Conceptualization, Methodology, Writing – review & editing.

## Declaration of competing interest

The authors declare that they have no known competing financial interests or personal relationships that could have appeared to influence the work reported in this paper.

## Figures and Tables

**Table:** 

	Cashew nut (n = 1)	Broad bean (n = 1)	Milk (n = 7)
*Energy and nutrients (mandatory to declare)*			
Energy (kcal)	23	49	50 (35, 70)
Total fat (g)	1.10	2.90	1.50 (0.05, 4.20)
Saturated fat (g)	0.20	0.20	1.00 (0.00, 2.70)
Carbohydrate (g)	2.60	3.60	4.80 (4.70, 9.80)
Total sugar (g)	2.00	2.40	4.90 (4.70, 8.80)
Protein (g)	0.50	2.10	3.50 (3.30, 3.60)
Salt (g)	0.13	0.06	0.10 (0.10, 0.20)
*Selected nutrients (non-mandatory to declare)*			
Fibre (g)^1^	0.20	N/A	0.00 (0.00, 0.50)
Vitamin D (mcg)^2^	0.75	1.50	1.00 (0.00, 1.00)
Vitamin B2 (mg)^3^	0.21	0.21	0.15 (0.14, 0.16)
Vitamin B12 (mcg)^4^	0.38	0.60	0.58 (0.10, 0.59)
Calcium (mg)^5^	120	120	120 (74, 124)
Iodine (mcg)^5^	N/A	N/A	12.00 (6.00, 25.80)

N/A: Data not available.

## Data Availability

Data will be made available on request.
